# Distribution and determinants of pneumonia diagnosis using Integrated Management of Childhood Illness guidelines: a nationally representative study in Malawi

**DOI:** 10.1136/bmjgh-2017-000506

**Published:** 2018-04-09

**Authors:** Omolara T Uwemedimo, Todd P Lewis, Elsie A Essien, Grace J Chan, Humphreys Nsona, Margaret E Kruk, Hannah H Leslie

**Affiliations:** 1 Department of Pediatrics and Occupational Medicine, Epidemiology and Prevention, Donald and Barbara Zucker School of Medicine at Hofstra/Northwell GLOhBAL (Global Learning. Optimizing health. Building Alliances Locally), Hempstead, New York, USA; 2 Department of Global Health and Population, Harvard TH Chan School of Public Health, Boston, New York, USA; 3 GLOhBAL (Global Learning. Optimizing health. Building Alliances Locally) at Cohen, Children’s Medical Center, New Hyde Park, New York, USA; 4 Malawi Ministry of Health (IMCI), Lilongwe, Malawi

**Keywords:** pneumonia, health systems, child health, cross-sectional survey

## Abstract

**Background:**

Pneumonia remains the leading cause of child mortality in sub-Saharan Africa. The Integrated Management of Childhood Illness (IMCI) strategy was developed to standardise care in low-income and middle-income countries for major childhood illnesses and can effectively improve healthcare worker performance. Suboptimal clinical evaluation can result in missed diagnoses and excess morbidity and mortality. We estimate the sensitivity of pneumonia diagnosis and investigate its determinants among children in Malawi.

**Methods:**

Data were obtained from the 2013–2014 Service Provision Assessment survey, a census of health facilities in Malawi that included direct observation of care and re-examination of children by trained observers. We calculated sensitivity of pneumonia diagnosis and used multilevel log-binomial regression to assess factors associated with diagnostic sensitivity.

**Results:**

3136 clinical visits for children 2–59 months old were observed at 742 health facilities. Healthcare workers completed an average of 30% (SD 13%) of IMCI guidelines in each encounter. 573 children met the IMCI criteria for pneumonia; 118 (21%) were correctly diagnosed. Advanced practice clinicians were more likely than other providers to diagnose pneumonia correctly (adjusted relative risk 2.00, 95% CI 1.21 to 3.29). Clinical quality was strongly associated with correct diagnosis: sensitivity was 23% in providers at the 75th percentile for guideline adherence compared with 14% for those at the 25th percentile. Contextual factors, facility structural readiness, and training or supervision were not associated with sensitivity.

**Conclusions:**

Care quality for Malawian children is poor, with low guideline adherence and missed diagnosis for four of five children with pneumonia. Better sensitivity is associated with provider type and higher adherence to IMCI. Existing interventions such as training and supportive supervision are associated with higher guideline adherence, but are insufficient to meaningfully improve sensitivity. Innovative and scalable quality improvement interventions are needed to strengthen health systems and reduce avoidable child mortality.

**Key questions**

**What is already known?**

- Pneumonia remains the leading cause of child mortality in sub-Saharan Africa.
- Studies from low-income and middle-income countries have demonstrated suboptimal clinical evaluation of pneumonia resulting in missed or delayed diagnosis and excess morbidity and mortality.

**What are the new findings?**

- This nationally representative study shows low guideline adherence and missed diagnosis for four out of every five Malawian children with pneumonia.
- Clinical quality and healthcare worker type were associated with diagnostic accuracy, but other contextual factors were not.

**What do the new findings imply?**

- Most potential pneumonia cases are missed despite widespread dissemination of standardised guidelines.
- Urgent consideration of methods not reliant on algorithmic guidelines and alternative quality improvement options that can be implemented at scale is required if low-income countries such as Malawi are to make further strides in child survival.

## Background

An estimated 6 million children under 5 years of age (‘under-fives’) still die each year worldwide; half of these deaths occur in sub-Saharan Africa.^1–3^ Pneumonia, in particular, remains the leading cause of under-five mortality outside of the neonatal period, accounting for one out of every six deaths.^3^ Moreover, the majority of deaths due to pneumonia are preventable with low-cost, proven interventions, including early identification and appropriate antibiotic treatment,^4^ yet only 58% of under-fives with symptoms of pneumonia, such as cough and fast breathing, are evaluated by a healthcare provider. Furthermore, research from sub-Saharan Africa has revealed that children often receive suboptimal assessments for pneumonia at health facilities, such as inadequate history-taking and physical examination, resulting in delayed or inadequate diagnosis and increased morbidity and mortality.^5 6^ Malawi is one of the poorest countries in sub-Saharan Africa, yet has made considerable progress in under-five survival over the past decades: mortality decreased from 273 per 1000 live births in 1990 to 71 by 2013.^7^ Notably, Malawi has demonstrated considerable success in scaling up community case management of childhood illness to extend the reach of the health system.^8^ In an effort to build on these gains, the Government of Malawi has identified priority conditions with considerable avertable mortality; nearly one in five child deaths from pneumonia could be averted with appropriate use of existing interventions.^9^

For over two decades and in over 100 countries, the Integrated Management of Childhood Illnesses (IMCI) has been a primary strategy to address deficiencies in case management of pneumonia in low-income, resource-limited settings, including in sub-Saharan Africa.^2 10^ IMCI was developed by WHO and United Nations Children’s Fund (Unicef) to assess and manage the major contributors to under-five mortality, including pneumonia, using stepwise algorithms for case management (appropriate assessment, classification, treatment and counselling).^2 11^ In sub-Saharan Africa, the IMCI protocol represents a ‘gold standard’ approach for standardised diagnosis of pneumonia in a region with reduced access to sophisticated diagnostic tools (eg, chest X-ray, pulse oximetry).^12^

Multiple studies have demonstrated IMCI as an effective strategy to improve quality of sick-child care and healthcare worker performance, and reduce childhood mortality.^10 13–20^ Despite proven *efficacy* of IMCI, the *effectiveness* of IMCI has been thwarted by poor adherence to IMCI algorithms during case management of pneumonia and other conditions.^21–24^ Major contributors to decreased adherence include shorter and less comprehensive training, limited availability of essential equipment and drugs, and lack of IMCI-specific routine supervision.^22^ Studies also suggest poor provider motivation and high workloads as explanations for low guideline adherence.^25^

Although pneumonia is the second largest contributor to global child mortality, few studies have investigated accuracy of provider assessment and classification of pneumonia using IMCI pneumonia classification guidelines.^5 21 24 26^ A review of pneumonia community case management identified poor adherence to guidelines and difficulty in identifying severe pneumonia as impediments to effective care in sub-Saharan Africa.^27^ A study of health surveillance assistance in Malawi similarly found that pneumonia was the least likely to be correctly identified and treated of common child illnesses.^28^ However, these studies report samples of sub-national data from select provinces, districts and/or health facilities, limiting exploration of factors such as the broader context of care that affect health worker performance in low-resource settings. Previous studies on assessment and management of pneumonia using nationally representative data have identified gaps in clinical assessment and poor prescribing practices. 29 30

We used data from a health facility census in Malawi to describe the quality of care received by children with symptoms of pneumonia and to determine the sensitivity of provider diagnosis of pneumonia among under-fives using IMCI criteria for pneumonia diagnosis. We then assessed potential determinants of correct diagnosis of pneumonia to identify opportunities for intervention to further reduce under-five mortality in Malawi.

## Methods

### Study sample

Data were obtained from the 2013–2014 Service Provision Assessment (SPA), a census of health facilities conducted by the Demographic and Health Survey programme. The SPA includes an audit of facility resources, surveys on clinical practices and direct observation of clinical care for children under the age of 5. In Malawi, the SPA also included direct re-examinations of sick children by trained survey personnel.

Data on the spatial distribution of poverty status were obtained through the WorldPop project, which uses Bayesian model-based geostatistics in combination with spatial covariates applied to GPS-located household survey data on poverty to generate estimates of individuals in poverty per square kilometre. The Malawi estimates are based on the 2010–2011 Living Standards Measurement Study programme and define extreme poverty as those living on US$1.25 or less per day.

### Outcome definition and assessment

Re-examinations of sick children included assessment of cough, respiratory rate (if cough was reported), anaemia, temperature and responsiveness. IMCI, which can be used by doctors, nurses and other health professionals to evaluate sick infants and children aged from 1 week to 5 years, has been established as a case management protocol for first-level facilities such as dispensaries, health centres or hospital outpatient departments.^11^ We used the IMCI Chart Booklet to define the symptoms that should result in a pneumonia diagnosis: for children from 2 to 59 months old, pneumonia is defined as either cough and stridor or cough and fast breathing (50+ breaths per minute for children 2 to 11 months old or 40+ breaths per minute for children 12 to 59 months old). These guidelines are in accordance with the Malawi national guidelines in place at the time of the survey.^31^ This analysis excluded children under 2 months old due to the absence of specific IMCI guidelines for pneumonia diagnosis in this age group. Given the information available from the re-examination, we defined pneumonia cases as children with cough and fast breathing on re-examination. Providers were asked the diagnosis they assigned following each patient visit as part of the direct clinical observation; results are thus not subject to potentially poor data quality of medical records. The primary study outcome is sensitivity of provider diagnosis of pneumonia, defined as the probability that a provider reported pneumonia as the child’s diagnosis if the child met the IMCI criteria on re-examination. Due to limited information from the re-examination, including no indication of stridor, we could not definitively identify children who did not meet the case definition of pneumonia; we thus did not assess specificity of diagnosis.

### Covariates

We identified multiple levels influencing sensitivity of pneumonia diagnosis: contextual factors such as poverty, facility characteristics, provider characteristics and child/visit characteristics. Poverty was calculated as percent of the population within 5 km of each facility living on less than US$1.25 per day (this calculation was performed in QGIS V.2.14; Free Software Foundation, Boston, Massachusetts, USA)). We categorised percent poverty into quintiles for descriptive analyses. Facility characteristics were defined as infrastructural and environmental factors that might be associated with diagnosis, such as location, facility management, facility type and structural quality, and provider factors such as professional level and technical quality score. Structural quality was measured with two indices of service readiness defined by WHO: general service readiness (50 indicators across five domains: basic amenities, basic equipment, infection prevention measures, diagnostic capacity and essential medications) and readiness for child preventive and curative care (18 indicators covering staff and training, basic equipment, diagnostics and medication for sick-child care).^32^ Technical quality was included in the analyses as the proportion of expected actions for all visits completed during a sick-child visit based on the IMCI Chart Booklet and the items available in the SPA dataset.^33^ Example items include history-taking (eg, provider asks whether the child has a normal feeding pattern), routine examination (eg, child is weighed), treatment (eg, child is given vitamin A) and client counselling (eg, provider provides directions for feeding to the caretaker). Analyses also included two variables regarding healthcare worker supports: training, defined as a healthcare worker reporting in-service training in IMCI within the past 2 years, and supportive supervision, defined as a healthcare worker reporting supervision in the last 6 months that included discussion of problems encountered and receipt of supervisor feedback. Child characteristics considered included child age (infant or not), sex and case severity, where severe case was defined as fever (temperature ≥37.5°C) and/or tachypnoea (respiratory rate ≥60 breaths per minute) as measured by a clinical observer.

### Statistical analysis

We calculated the sensitivity of pneumonia diagnosis for each age group by assessing the number of clinician-diagnosed pneumonia cases among all IMCI-determined pneumonia cases in a series of 2×2 contingency tables.

All subsequent analyses were performed within the children with IMCI-determined pneumonia. Among children who met the case definition for pneumonia but did not receive a pneumonia diagnosis, we assessed the proportion who received common alternative diagnoses. We calculated level of sensitivity across the binary and categorical covariates and tested significance of any differences using F tests corrected for the design effect of repeated sampling within facility. We then compared average structural and technical quality between children who were correctly and incorrectly diagnosed, determining significance using F tests again clustered by facility.

We constructed multivariable models to test the association of the key covariates at each of the levels defined above with sensitivity, controlling for confounding by higher-level factors. Model 1 tested broad facility factors: location (urban or not), management type and facility type; we consolidated facility types into hospitals and non-hospitals following the tiers of the health system in Malawi. Model 2 assessed facility-specific structural readiness. Model 3 and Model 4 incorporated provider capacity, including provider cadre and the technical quality of the care received, respectively. The final model assessed healthcare worker supports, including recent training in IMCI and supportive supervision. We identified child characteristics as factors that might be associated with pneumonia diagnosis, but that should not affect facility or visit characteristics; we tested each for inclusion as a control variable in unadjusted models with sensitivity using a criterion of P <0.20. We conducted a similar analysis to select contextual factors such as poverty and urban versus rural location due to the small sample size, controlling only for those related to the outcome at P <0.20. We employed log-binomial generalised estimating equation (GEE) models clustered by facility to calculate the relative risk (probability) of correct diagnosis; GEE models provide robust SEs to account for non-independence of observations within facilities. In instances where this model form failed to converge, we used GEE models with a Poisson distribution and robust SEs, which estimates the adjusted incidence rate ratio to approximate the relative risk.^34^ We predicted estimated sensitivity at observed levels of key factors such as technical quality while holding all other variables at their means.

We conducted two additional analyses to contextualise the main analyses. To test whether technical quality was differentially associated with sensitivity in better-equipped facilities, we assessed the association between technical quality and sensitivity of diagnosis only for children who received care in facilities with above-average structural quality for child care. We also explored the potential impact of training in IMCI and supportive supervision on sensitivity by comparing technical quality between providers reporting in-service training in IMCI in the past 2 years, supportive supervision in the last 6 months or both, and those without such supports. We predicted sensitivity if all providers provided the level of technical quality observed among providers with IMCI training, supportive supervision or both.

For descriptive analyses of the full sample (table 1), child characteristics were weighted by the SPA client sampling weight, and facility characteristics were weighted by the SPA facility sampling weight, rescaled to the analytic sample. All other analyses, which pertain to the subsample of children with pneumonia symptoms, are unweighted. All analyses were conducted using Stata/SE V.14.2.

**Table 1:** Study sample description: characteristics of facilities providing sick-child care and children receiving care

Variable	N	%	N	%
	Children under 5 receiving sick-child care (n=3248)		Facilities providing sick-child care (n=742)	
Client demographics				
Child sex				
Female	1603	49		
Child age (months)				
<2 months	112	3		
2 through 11 months	1060	33		
12 through 59 months	2076	64		
Symptoms reported by caretaker*				
Cough	320	10		
Fever	130	4		
Cough+fever only	505	16		
Cough+fever+other symptom†	1069	33		
Cough+other symptom, no fever	445	14		
Fever+other symptom, no cough	455	14		
Other symptom (no cough or fever)	267	8		
No symptoms reported by caretaker	58	2		
Clinician characteristics				
Qualification of clinician caring for sick child (child level)/highest clinician on site (facility level)				
Physician	35	1	90	12
Advanced practice clinician or paramedical professional (eg, assistant medical officer, clinical officer)	2771	85	593	80
Nurse	438	13	55	7
Other health professional (eg, counsellor, social worker)	4	1	3	1
In-service training and supportive supervision				
IMCI training in the past 6 months: clinician caring for sick child (child level)/at least one clinician on site (facility level)	539	17	217	29
Supportive supervision in the past 6 months: clinician caring for sick child (child level)/at least one clinician on site (facility level)‡	1881	58	458	62
Facility characteristics				
Poverty in facility catchment area				
>80% in extreme poverty	202	6	57	8
60–80% in extreme poverty	1428	44	348	47
40–60% in extreme poverty	1202	37	228	31
20–40% in extreme poverty	207	6	57	8
0–20% in extreme poverty	209	6	48	7
Urban/non-urban				
Urban	1023	32	181	24
Public/private				
Private	780	24	330	45
Type of facility				
Hospital	1167	36	98	13
Non-hospital (eg, health centre, clinic, dispensary)	2081	64	644	87
Quality scores	Mean	SD	Mean	SD
Structural quality				
Service Readiness Index§	0.64	0.17	0.59	0.14
Service readiness for child curative and preventive care¶	0.65	0.16	0.58	0.16
Technical quality				
Observed adherence to IMCI**	0.30	0.13	0.31	0.11

*Caretakers could report multiple symptoms for each child.

†Other symptoms include diarrhoea, vomit, feeding problem, convulsions, sleeping problem and other.

‡Supportive supervision is defined as supervision that included feedback and discussion of problems encountered in the past 6 months.

§Service Readiness Index is a score from 0 to 1 assessing facility preparedness to deliver healthcare based on 50 items in five domains: amenities, basic equipment, infection prevention, diagnostic capacity and essential medicine (WHO SARA report).

¶Service readiness for curative and preventive care for children is the proportion of 18 items (eg, staff training in IMCI, a thermometer and amoxicillin) essential for these services (WHO SARA report).

**Observed adherence to IMCI guidelines is the proportion of clinical actions (eg, history, examination, management) the provider is observed to complete in each clinical visit out of a list of 22 items (17 for those <2 months) detailed in the IMCI guidelines (2014 Chartbook).

IMCI, Integrated Management of Childhood Illness.

### Ethical approval

The original survey implementers obtained ethical approvals for data collection from the National Health Science Research Committee, Ministry of Health, Malawi and the Institutional Review Board at ICF International. The Harvard University Human Research Protection Program deemed this analysis exempt from human subjects review (protocol no. IRB16-1941).

## Results

The SPA assessed 977 of 1066 health facilities in Malawi (92% response rate); 3% of facilities refused assessment, while the remainder were closed, empty or inaccessible.^35^ Of these, 918 provided curative care to children, and 742 had at least one direct observation of child care. In total, care was observed for 3248 children under 5 years of age; 112 of these were children under 2 months who were excluded from analysis.

Table 1 describes characteristics of facilities providing sick-child care and children receiving care. Children in the sample were 51% male and 49% female, with 64% from 12 to 59 months of age. The most common symptom set reported by the child’s caretaker was cough with fever and an additional symptom (eg, diarrhoea, vomit, feeding problem, convulsions or sleeping problem) (33%), or cough and fever alone (16%). Children most frequently received care from advanced practice clinicians or paramedical professionals, such as assistant medical officers or clinical officers, in public facilities in rural areas. The majority of sick-child visits (61%) occurred at non-hospitals, such as health centres or clinics, in areas where the proportion of individuals in extreme poverty was between 40% and 80%. Most facilities were publicly owned (55%), in non-urban areas (76%). Considerable gaps were evident in structural capacity to provide care: facilities scored an average of just under two-thirds on both general service readiness and child-specific service readiness. Average technical quality was very low: 30% (SD 0.13). Online supplementary figure A1 shows the performance of each IMCI item across visits; providers assessed history of cough in 74% of visits and took temperature two-thirds of the time, but assessed respiratory rate in only 16% of visits.

10.1136/bmjgh-2017-000506.supp1 Supplementary data

Of the 3136 children in our sample at least 2 months old, 573 (18%) met the IMCI-based criteria for pneumonia diagnosis based on re-examination. Only 118 children (21%) of these children were correctly diagnosed with pneumonia (table 2). Of the 455 children meeting the case definition of pneumonia who were not diagnosed with it (ie, had a ‘missed diagnosis’), 212 (46%) were diagnosed with an upper respiratory infection and 158 (34%) were diagnosed with malaria; older children were more likely to be diagnosed with malaria than children under one (36.4% vs 21.4%, P<0.001, data not shown). Among the children meeting IMCI criteria for pneumonia, the overwhelming majority (90%) had symptoms with a duration of less than 1 week, suggesting acute illness (data not shown). Sensitivity was lower in children 12 months to 59 months old, with 78 of 428 (18% sensitivity) diagnosed with pneumonia compared with 40 of 145 cases (28% sensitivity) in children under one. Healthcare workers were more likely to assign the correct diagnosis when children presented with fever, high respiration or both as well as cough: sensitivity among these children (n=206) was 28%. Although prevalence of confirmed fever was equal across age groups, caretakers of children over one were more likely to report fever as a cause for the visit (76.7% vs 63.4%, P=0.003), with or without cough or other symptom (data not shown).

**Table 2:** Diagnosis of pneumonia versus IMCI case definition in children 2–59 months old in Malawi (n=3136)

Test: clinician diagnosis	IMCI Dx+	IMCI Dx−	
A. All children 2 months through 59 months			
Clinician Dx+	118	241	359
Clinician Dx−	455	2322	2777
	573	2563	3136
Sensitivity	20.6%		
B. Children 12 months through 59 months			
Clinician Dx+	78	122	200
Clinician Dx−	350	1526	1876
	428	1648	2076
Sensitivity	18.2%		
C. Children 2 months through 11 months			
Clinician Dx+	40	119	159
Clinician Dx−	105	796	901
	145	915	1060
Sensitivity	27.6%		

IMCI, Integrated Management of Childhood Illness.

Sensitivity was low across the range of included covariates (table 3). Among client demographics and facility characteristics, sensitivity of diagnosis was significantly higher for children <12 months of age, those with fever and/or high respiration, and those seen by clinicians as compared with nurses and other providers. Children seen in dispensaries were less likely to be correctly diagnosed than those seen in hospitals, health centres and clinics; the difference did not reach statistical significance. Analysis of case severity using presence of fever and/or high respiration did not identify differences in the case mix presenting by facility type or clinician qualification (see online supplementary table A1). Average technical quality was significantly higher for children receiving a correct diagnosis of pneumonia (0.38 vs 0.31), but structural quality and child-specific structural quality did not differ significantly between those correctly and incorrectly diagnosed.

**Table 3:** Association of child, clinician and facility characteristics with provider diagnosis of pneumonia among children with pneumonia by IMCI guidelines (n=573)

Variable	Sensitivity (%)	P value F test	N
Client demographics			
Child age (months)			
2 through 11 months	28%		145
12 through 59 months	18%	0.019	428
Fever and/or tachypnoea confirmed by re-examination			
Severe case: child has fever and/or tachypnoea	28%		206
Less severe case: child has neither fever nor tachypnoea	16%	0.001	367

Clinician characteristics			
Qualification of clinician providing care for sick-child visit			
Physician, advanced practice clinician or paramedical professional (eg, assistant medical officer, clinical officer)	23%	0.025	457
Nurse or other health professional (eg, counsellor, social worker)	13%		116
In-service IMCI training			
Training in past 6 months	23%	0.650	83
Training in past 6 months No recent training	20%		475
Supportive supervision			
Supportive supervision in past 6 months	22%	0.300	354
No supportive supervision	18%		219

Facility characteristics			
Poverty in facility catchment area			
>80% in extreme poverty	22 %	0.922	37
60–80% in extreme poverty	20%		312
40–60% in extreme poverty	23%		164
20–40% in extreme poverty	17%		36
0–20% in extreme poverty	21%		24
Urban/non-urban			
Urban	15%	0.114	107
Non-urban	22%		466
Public/private			
Private	22%		190
Public	20%	0.552	383
Type of facility			
Hospital	18%		87
Health centre	23%		371
Clinic	18%	0.228	87
Dispensary	7%		28

Quality scores	Mean quality by provider diagnosis of pneumonia (SD)		P value F test
	Yes	No	
Structural quality			
Service Readiness Index	0.59 (0.14)	0.59 (0.16)	0.968
Service readiness for child curative and preventive care	0.61 (0.14)	0.60 (0.17)	0.522
Technical quality			
Observed adherence to IMCI	0.38 (0.13)	0.31 (0.12)	<0.001

IMCI, Integrated Management of Childhood Illness.

Child age above and below 1 year, case severity and urban versus rural location met our screening criterion but child sex and poverty did not; we thus controlled for age, severity and location in all models. As shown in column 2 of table 4, probability of correct diagnosis among children with pneumonia symptoms was higher in rural areas and privately managed facilities, but not significantly so. Sensitivity did not vary significantly by general and child-specific structural quality (model 2). In model 3, clinicians were twice as likely to provide the correct diagnosis to children with pneumonia symptoms as nurses and other providers (adjusted relative risk 2.00, 95% CI 1.21 to 3.29). Technical quality was strongly associated with sensitivity of pneumonia diagnosis with an adjusted incidence rate ratio (AIRR) of 18.05 (95% CI 5.48 to 59.46). In the final model, neither receipt of IMCI training nor supportive supervision was significantly associated with sensitivity of pneumonia diagnosis. Holding all other factors at their mean, providers at the 75th percentile of technical quality in the sample (41% adherence to IMCI guidelines) are estimated to correctly diagnose 23% of children with pneumonia compared with 14% correct diagnosis for providers at the 25th percentile (23% adherence). The association between technical quality and sensitivity was similar when limited to facilities with above-average structural readiness for sick-child care (AIRR 16.9, results not shown).

**Table 4:** Multivariable regressions on sensitivity of pneumonia diagnosis among symptomatic children (n=573)

	Model 1	Model 2	Model 3	Model 4	Model 5
	ARR (95% CI)	ARR (95% CI)	ARR (95% CI)	AIRR (95% CI)	ARR (95% CI)
Urban	0.63 (0.39 to 1.04)	0.63 (0.38 to 1.04)	**0.59 (0.36 to 0.97)**	0.73 (0.44 to 1.19)	**0.59 (0.36 to 0.99)**
Private	1.19 (0.85 to 1.67)	1.16 (0.81 to 1.66)	1.33 (0.92 to 1.92)	1.18 (0.81 to 1.73)	1.34 (0.92 to 1.96)
Hospital	1.00 (0.61 to 1.66)	0.87 (0.47 to 1.62)	0.80 (0.44 to 1.46)	0.80 (0.46 to 1.38)	0.92 (0.48 to 1.77)
General service readiness		1.42 (0.28 to 7.16)	1.16 (0.24 to 5.64)	0.86 (0.17 to 4.33)	0.97 (0.18 to 5.20)
Child service readiness		1.33 (0.34 to 5.15)	1.39 (0.37 to 5.16)	1.57 (0.41 to 5.99)	1.32 (0.33 to 5.12)
Clinician (vs nurse/other)			**2.00 (1.21 to 3.29)**	**1.91 (1.15 to 3.18)**	**2.00 (1.21 to 3.31)**
Technical quality				**18.05 (5.48 to 59.46)**	
IMCI training					1.03 (0.63 to 1.68)
Supportive supervision					1.13 (0.80 to 1.60)
Child age ≥1 year	0.80 (0.57 to 1.13)	0.79 (0.56 to 1.12)	0.80 (0.57 to 1.12)	0.78 (0.56 to 1.08)	0.76 (0.55 to 1.06)
Severe case: fever and/or tachypnoea	**1.59 (1.17 to 2.17)**	**1.60 (1.17 to 2.17)**	**1.58 (1.17 to 2.15)**	**1.55 (1.16 to 2.09)**	**1.61 (1.19 to 2.19)**

Boldface type used for associations found to be significant at P <0.05. Clinician includes clinical officers, assistant medical officers and medical officers; other includes counsellors and social workers. Service readiness is an index from 0 to 1 comprising basic amenities, equipment, infection prevention, diagnostics and medications. Child service readiness is an index from 0 to 1 comprising staff and training, guidelines, and child-specific equipment and medication. Technical quality is the adherence to IMCI guidelines for clinical visits expressed as a proportion from 0 to 1.

AIRR, adjusted incidence rate ratio; ARR, adjusted relative risk; IMCI, Integrated Management of Childhood Illness.

Providers who had a recent training on IMCI (14% of providers; see online supplementary appendix table A2) had an average technical quality score of 35% as compared with 30% for providers without a recent training; those receiving supportive supervision (59% of providers) showed 32% adherence to IMCI guidelines compared with 29% among those not supervised. If all healthcare workers adhered to clinical guidelines as well as those recently trained in IMCI, those supervised or both, predictions based on the adjusted regression (model 4) suggest that the relatively small difference in technical quality would be insufficient to increase sensitivity of pneumonia diagnosis beyond the current level of 21%.

**Figure 1:**
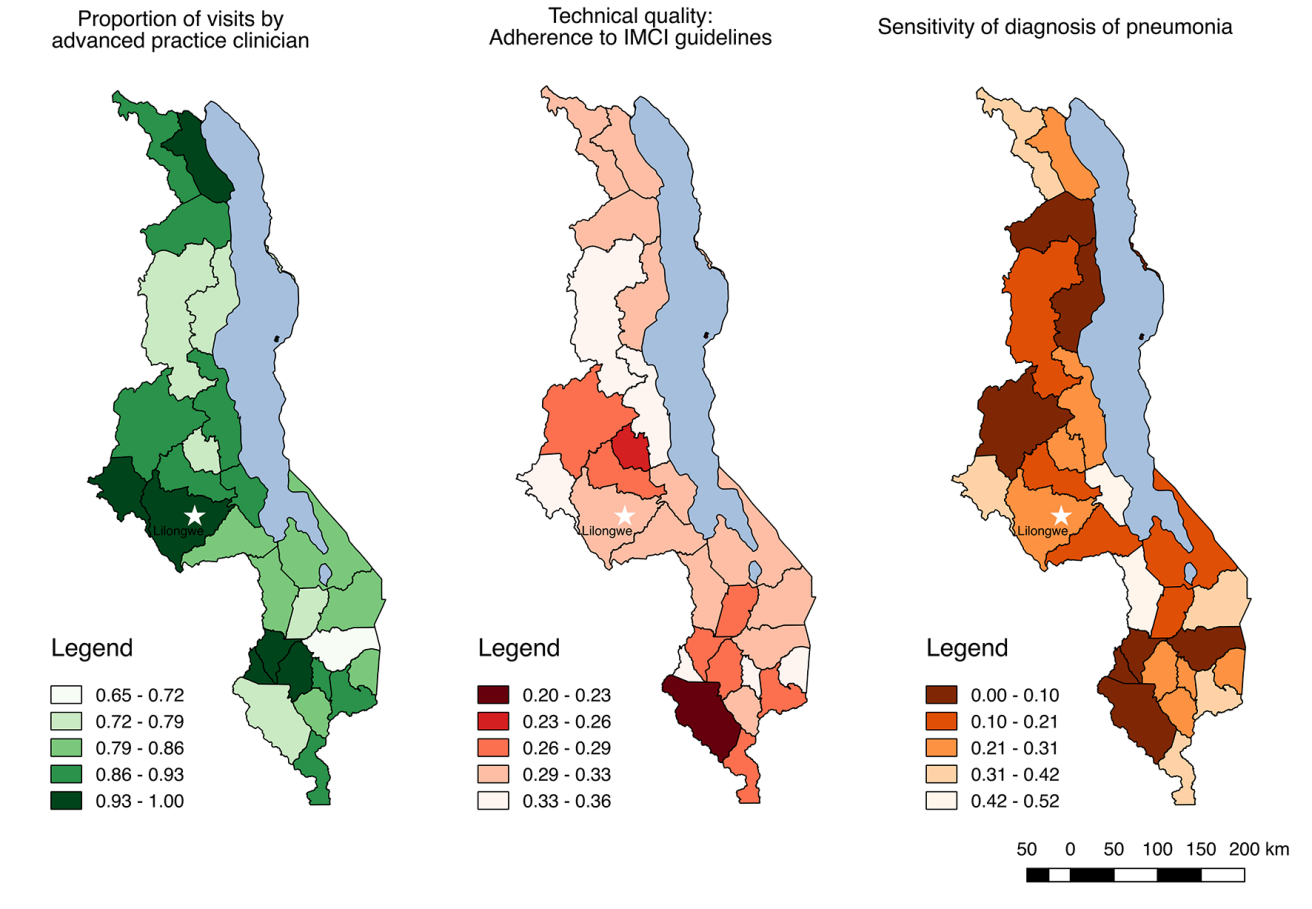
Technical quality of care, sensitivity of pneumonia diagnosis and proportion of visits by advanced practice clinicians for sick children by district in Malawi. IMCI, Integrated Management of Childhood Illness.

Figure 1 maps technical quality and sensitivity of pneumonia diagnosis by district in Malawi; the results underscore the widespread distribution of poor quality despite the majority of visits taking place with a physician or advanced practice clinician, with lower than 50% adherence to IMCI guidelines and a higher probability of missed diagnosis than correct diagnosis in all but one district.

## Discussion

To our knowledge, this study is the first to examine sensitivity of provider diagnosis of pneumonia using a nationally representative sample of patients across all districts and health facility types within a low-income country. Consistent with prior studies in low-income and middle-income countries, we found low diagnostic accuracy of pneumonia, with providers in Malawi identifying only one in five pneumonia cases according to IMCI diagnostic criteria among children aged 2 to 59 months. Missed diagnoses were common irrespective of surrounding poverty level, urban versus rural location and facility type. Providers under-diagnosed pneumonia at both well-equipped and poorly equipped facilities. Higher sensitivity was associated with provider factors, specifically provider type and higher adherence to IMCI, but clinicians and those more adherent to clinical guidelines still missed more cases than they correctly identified. This evidence suggests a critical quality gap in formal healthcare services in Malawi, one that contributes to excess morbidity and avertable child deaths.

Patient factors, such as child age and presenting with fever and/or tachypnoea, were associated with correct diagnosis in bivariable analysis. This is consistent with previous research^19 36^ and may suggest a lower threshold for diagnosing pneumonia in infants, who experience higher pneumonia incidence and mortality.^37 38^ In this study, caretakers of older children were more likely to report fever among the presenting complaints; these children were more likely than infants to be diagnosed with malaria. Healthcare providers may be predisposed to look for pneumonia in infants and malaria in older children; given the incompleteness of physical examinations conducted (five of six children did not have respiratory rate assessed), caretaker report and expected prevalence of disease may form much of the basis for diagnosis. Higher sensitivity among children with fever or tachypnoea suggests providers are responsive to case severity, though three of four children with indications of severe illness were not diagnosed with pneumonia.

While few factors were associated with correct diagnosis of pneumonia, provider cadre was a significant determinant, with nurses and other providers demonstrating lower sensitivity compared with clinicians (eg, clinical officers, physicians). Due to massive shortages of physicians in sub-Saharan Africa, nurses compose the majority of providers available for sick-child care at first-level health facilities accessible to rural communities, families in extreme poverty and individuals with limited transport.^39 40^ Task-shifting implemented in response to the health workforce shortage has resulted in the delegation of clinical responsibilities to existing or new cadres with either less training or narrowly tailored training.^41^ Although nurses in first-line facilities encounter multiple time and material constraints in performing assessments according to IMCI guidelines,^42^ our results suggest that the difference between clinicians and nurses remained after controlling for facility type and resources available. These findings call into question the effectiveness of non-clinician healthcare workers in appropriately identifying pneumonia cases. Still, even with increased physician or advanced practice clinician presence, sensitivity of pneumonia diagnosis among these providers remains extremely low.

As the first nationally representative study on accuracy of pneumonia diagnosis in a low-income country, these results reinforce existing signals of a quality crisis. Prior analysis of the SPA data in Malawi found that nearly 30% of children who needed antibiotics did not receive them, while nearly 60% of children without antibiotic need were prescribed them.^29^ Our results confirm low rates of appropriate assessment such as respiratory rate identified in an analysis of these data, with particularly weak performance among children over 1.^30^ Similarly, a subnational assessment in Lilongwe found 30% correct diagnosis of pneumonia by clinical officers in Lilongwe, with respiratory rate assessed in only 16% of patients.^5^ A recent study of 161 febrile children in Zambia found that no health workers performed a complete assessment or physical examination; respiratory rate assessment was rare and only half of those diagnosed with pneumonia were correctly treated.^43^ Broader studies on adherence to IMCI guidelines support the findings here of only 30% completion: a comparative assessment across seven countries estimated that providers completed only half of evidence-based actions during an average sick-child visit,^44^ while meta-analyses and reviews have identified similarly poor performance.^45 46^ One exception is a smaller study of 23 clinicians in two districts of Malawi, which found closer to 70% adherence to IMCI guidelines.^12^ The government of Malawi has demonstrated considerable commitment to improving population health, with demonstrable success in increasing access to care and reducing mortality.^7^ Existing health sector plans acknowledge the importance of improving care quality.^9^ This evidence adds urgency to the task of identifying and implementing effective, scalable quality improvement programmes.

While reinforcing the need for quality improvement, this study demonstrates mixed results for common strategies. IMCI technical quality predicted sensitivity of pneumonia diagnosis: above average providers correctly identified an estimated 10% more cases of pneumonia. However, the low baseline adherence raises the question of whether and how provider performance can be increased enough to meaningfully improve diagnostic accuracy. The two most prominent interventions—in-service training and supportive supervision—showed only a modest impact on provider adherence to guidelines. IMCI training has long been the intervention of choice for improving IMCI performance, though implementation and evaluation studies have identified significant heterogeneity of the effects on quality of care, including illness classification.^45^ While providers who had received recent training were more adherent to IMCI guidelines, this difference was insufficient to increase expected sensitivity beyond 25%. As found in prior research in multiple countries, the clinical impact of training appears to be minimal,^47^ equating to only 1.5 more clinical actions for trained providers compared with providers without recent training. The weak effects of training may stem from provision of modified training—shorter duration and omitting key components such as follow-up supervision—that is associated with only marginal improvements in clinical performance.^24^ Similarly, health services research in low-income countries consistently recognises supervision as a mainstay for improving provider performance.^26 48 49^ Effective and supportive supervisory staff have the capacity to impact healthcare workers in a number of ways: defining clinical goals, monitoring performance, analysing and reporting patient outcomes, increasing motivation and job satisfaction, and assisting with problem solving and planning.^50^ Although supportive supervision was common in this study, supervision was not associated with increased sensitivity in provider diagnosis or meaningful differences in technical quality. This may be due in part to inadequate supervisor training in conducting observational visits, chart audits, giving constructive feedback and providing individualised support for providers.^39 50^ Combined with prior research, our findings suggest that in-service training and supportive supervision as currently delivered are insufficient to boost and sustain quality of IMCI care. Lastly, existing qualitative research has highlighted poor adherence to IMCI, including suboptimal belief in the importance of adherence and lack of capacity to use IMCI algorithms for every case due to patient volume. In light of this, clinicians reported using ‘simpler rules of thumb’ for assessment and diagnosis and receiving insufficient remuneration to incentivise use of IMCI in lieu of their traditional clinical training.^25^

A second, complementary approach includes multidisciplinary care teams^51^ or ongoing mentorship programmes using trained nurse mentors.^52 53^ Implementation trials have identified timely clinical audits as a means of strengthening accountability for application of IMCI, but these required considerable investment.^49 54^ Efforts to integrate quality improvement activities into the institutional culture of health facilities offer potential for sustainable improvement such as the use of quality improvement packages in reproductive health services.^55 56^ Supportive supervision may be strengthened and standardised through tools such as the electronic Tool to Improve Quality of Healthcare (e-TIQH) piloted in Tanzania.^55^ Formative supervision, which combines observation and individualised, immediate feedback with a problem-solving approach to health service delivery, also offers promise.^53 57 58^ Integrating innovative supervision tools may also decrease the degree by which providers’ actions in practice deviate from what they know.^59 60^ The challenge facing quality improvement researchers is to demonstrate the scalability of such solutions to address broad and systematic deficits in quality like those in this study.

Looking beyond the current common interventions, multiple strategies emerge as potential approaches to delivering better quality care. One is to invest in health systems innovations such as mobile health applications and pulse oximetry, reducing provider dependence on adherence to the algorithmic IMCI guidelines.^31 61–65^ Research in Malawi on the feasibility of pulse oximetry demonstrates reasonable reliability among health workers and successful identification of children with hypoxia who would not have been recognised using clinical guidelines alone.^31^ Not all technological fixes are warranted: while rapid pneumonia tests have proven useful in guiding treatment decisions in adults, their poor specificity in children due to high rates of asymptomatic *Streptococcus pneumoniae* renders them inadequate for addressing childhood illness.^66^

Data used in this study were based on a nationally representative sample of children brought for sick-child care; reported symptoms and provider diagnosis were obtained directly from the caretaker and provider, eliminating error due to incomplete medical records. Standard diagnosis was determined based on re-examination by a trained observer. However, the implications of this study are subject to limitations. Based on the brief nature of the re-examination, we were able to consider only pneumonia; we could not completely rule out additional diagnoses and were not able to assess sensitivity of diagnoses of common conditions such as malaria, limiting the generalisability of these findings to other illnesses. However, we used all available data to consider the possibility of alternative diagnoses among children meeting the IMCI definition of pneumonia, and the short duration of symptoms in the majority of cases suggests that at least tuberculosis is an unlikely alternative diagnosis. In the future, complete assessment by a highly trained clinician would serve as a better standard for diagnostic accuracy of multiple conditions. We were unable to calculate specificity due to insufficient information to define those not meeting the case definition of pneumonia, although IMCI guidelines are intended to serve as a highly sensitive means of identifying severe illness, making sensitivity a more critical indicator than specificity. Assessment of interventions such as training relied on the details provided in the health facility assessment, which did not include the duration or specific format of the training or the qualifications of the supervisor.

## Conclusions

Despite the implementation of IMCI guidelines across Malawi more than a decade ago^8^ and significant gains in access to care, quality of care remains poor: children at high risk for morbidity and mortality were incorrectly diagnosed by formal healthcare providers throughout the country. The most common quality interventions—supportive supervision and in-service training—appear ineffective in significantly improving sensitivity of provider diagnosis of pneumonia in children. In Malawi and throughout low-income countries confronting a quality crisis, consideration of methods not reliant on algorithmic guidelines and alternative quality improvement options that can be implemented at scale is warranted. Effective strategies in improving quality of pneumonia care, as well as case management of other major causes of child mortality, are urgently needed if Malawi is to improve child survival.
