# Correction: *Distribution and determinants of pneumonia diagnosis using integrated management of childhood illness guidelines: a nationally representative study in Malawi*

**DOI:** 10.1136/bmjgh-2017-000506corr1

**Published:** 2018-06-15

Uwemedimo OT, Lewis TP, Essien EA, *et al*. Distribution and determinants of pneumonia diagnosis using integrated management of childhood illness guidelines: a nationally representative study in Malawi. *BMJ Glob Health* 2018;3:e000506. doi: 10.1136/bmjgh-2017-000506.

This article has been corrected since it first published. Owing to a communication lapse within the author team, the authors were not aware that findings from a related article that included co-author Humphreys Nsona were published while this article was under review and could be cited. This article focused on management of pneumonia cases once diagnosed. In addition, a typographic error resulted in the omission of an additional citation. The corrections are:

- Addition to Introduction, paragraph 4: ’Previous studies on assessment and management of pneumonia using nationally representative data have identified gaps in clinical assessment and poor prescribing practices.^[29,30]'^
- Inclusion of missing citation, Discussion paragraph four at end of sentence, ’Prior analysis of the SPA data in Malawi found that nearly 30% of children who needed antibiotics did not receive them, while nearly 60% of children without antibiotic need were prescribed them.’
- Addition to Discussion, paragraph 4: ’Our results confirm low rates of appropriate assessment such as respiratory rate identified in an analysis of these data, with particularly weak performance among children over 1.^[30]'^

The references are:

[29] Johansson EW, *et al*. Integrated paediatric fever management and antibiotic over-treatment in Malawi health facilities: data mining a national facility census.’ *Malar J* 2016;15:396.

[30] Johannson EW, *et al*. Determinants of Integrated Management of Childhood Illness (IMCI) non-severe pneumonia classification and care in Malawi health facilities: Analysis of a national facility census. *J Global Health* 2017;7.

