# Supplementary material for: Distribution and determinants of pneumonia diagnosis using Integrated Management of Childhood Illness guidelines: a nationally representative study in Malawi
Source: BMJ Glob Health. 2018 Apr 9;3(2):e000506. doi: 10.1136/bmjgh-2017-000506 (PMC5898357; doi:10.1136/bmjgh-2017-000506)
Supplement: Supplementary data [file bmjgh-2017-000506supp001.pdf]

APPENDIX

Figure A1: Provider adherence to IMCI guidelines (N=3248 clinical observations)

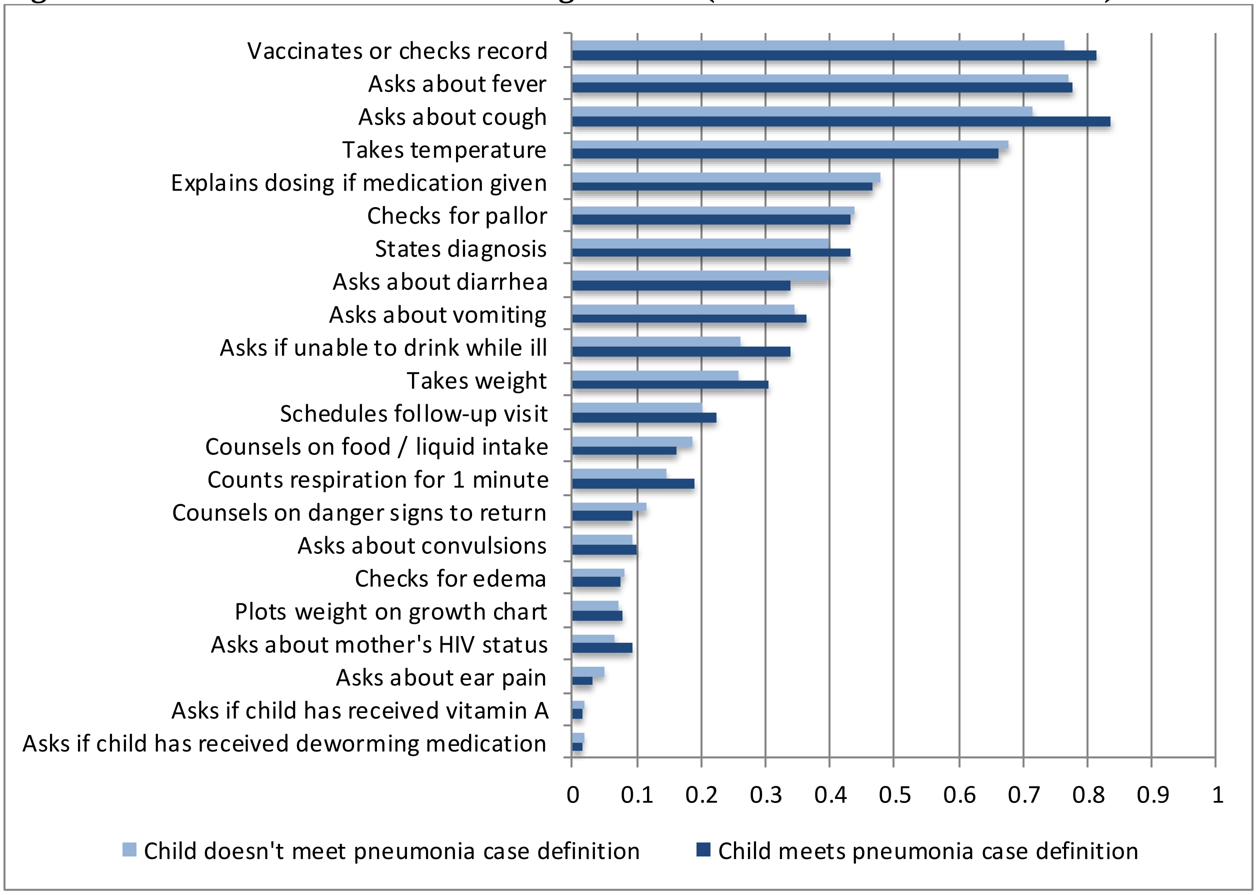

Table A1: Case mix among children with IMCI-diagnosed pneumonia by facility and clinician qualification (N=573)[1]

A. Facility type

	Non-hospital	Hospital	Total
	N (%)	N (%)	
Less severe case	310 (64)	57 (66)	367
Severe case	176 (36)	30 (34)	206
	486	87	573

B. Clinician qualification

	Nurse/other	Clinician	Total
	N (%)	N (%)	
Less severe case	74 (64)	293 (64)	367
Severe case	42 (36)	164 (36)	206
	116	457	573

[1]Case severity defined as child presenting with fever (temperature of 37.5 C or higher) and/or tachypnea (respiratory rate of 60 breaths per minute or higher) determined on re-examination

Table A2: Recent training on IMCI and supportive supervision among health care workers providing sick child care

Health care worker cadre	Total providers (N = 808)	Received support N (%)	%
In-service training in IMCI in past 2 years			
Physician (MD or medical officer)	18	1	6
Advanced practice clinician	8	0	0
Paramedical professional	630	98	16
Nurse (RN/RM, PHN/PHM)	7	0	0
Enrolled nurse/midwife	134	14	10
Other nurse/midwife	7	0	0
Other (counselor, social worker)	3	3	100
Supportive supervision in past 6 months			
Physician (MD or medical officer)	18	5	26
Advanced practice clinician	8	4	52
Paramedical professional	630	380	60
Nurse (RN/RM, PHN/PHM)	7	2	28
Enrolled nurse/midwife	134	85	63
Other nurse/midwife	7	3	43
Other (counselor, social worker)	3	0	0
Both training and supervision			
Physician (MD or medical officer)	18	1	6
Advanced practice clinician	8	0	0
Paramedical professional	630	69	11
Nurse (RN/RM, PHN/PHM)	7	0	0
Enrolled nurse/midwife	134	10	8
Other nurse/midwife	7	0	0
Other (counselor, social worker)	3	0	0

Percents reflect weighted averages
